# Dynactin Deficiency in the CNS of Humans with Sporadic ALS and Mice with Genetically Determined Motor Neuron Degeneration

**DOI:** 10.1007/s11064-013-1160-7

**Published:** 2013-09-28

**Authors:** Magdalena Kuźma-Kozakiewicz, Agnieszka Chudy, Beata Kaźmierczak, Dorota Dziewulska, Ewa Usarek, Anna Barańczyk-Kuźma

**Affiliations:** 1 Department of Neurology, Medical University of Warsaw, Warsaw, Poland; 2 Department of Biochemistry, Medical University of Warsaw, Banacha 1, 02-097 Warsaw, Poland; 3 Neurodegenerative Diseases Research Group, Medical University of Warsaw, Warsaw, Poland; 4 Department of Experimental and Clinical Neuropathology, Mossakowski Medical Research Institute, Polish Academy of Sciences, Warsaw, Poland

**Keywords:** Amyotrophic lateral sclerosis, Motor neuron degeneration, Dynactin, Retrograde axonal transport, Transgenic mice, hSOD1G93A mutation, *Dync1h1* mutation

## Abstract

Dynactin is a complex motor protein involved in the retrograde axonal transport disturbances of which may lead to amyotrophic lateral sclerosis (ALS). Mice with hSOD1G93A mutation develop ALS-like symptoms and are used as a model for the disease studies. Similar symptoms demonstrate Cra1 mice, with *Dync1h1* mutation. Dynactin heavy (DCTN1) and light (DCTN3) subunits were studied in the CNS of humans with sporadic ALS (SALS), mice with hSOD1G93A (SOD1/+), *Dync1h1* (Cra1/+), and double (Cra1/SOD1) mutation at presymptomatic and symptomatic stages. In SALS subjects, in contrast to control cases, expression of DCTN1-mRNA but not DCTN3-mRNA in the motor cortex was higher than in the sensory cortex. However, the mean levels of DCTN1-mRNA and protein were lower in both SALS cortexes and in the spinal cord than in control structures. DCTN3 was unchanged in brain cortexes but decreased in the spinal cord on both mRNA and protein levels. In all SALS tissues immunohistochemical analyses revealed degeneration and loss of neuronal cells, and poor expression of dynactin subunits. In SOD1/+ mice both subunits expression was significantly lower in the frontal cortex, spinal cord and hippocampus than in wild-type controls, especially at presymptomatic stage. Fewer changes occurred in Cra1/SOD1 and Cra1/+ mice.It can be concluded that in sporadic and SOD1-related ALS the impairment of axonal retrograde transport may be due to dynactin subunits deficiency and subsequent disturbances of the whole dynein/dynactin complex structure and function. The *Dync1h1* mutation itself has slight negative effect on dynactin expression and it alleviates the changes caused by SOD1G93A mutation.

## Introduction

Amyotrophic lateral sclerosis (ALS) is one of the most devastating, incurable neurodegenerative disease characterized by a progressive and selective loss of motor neurons [1]. Being the 4th most frequent neurodegenerative disease (after AD, FTD and PD), ALS is responsible for 1 for every 1,000 deaths worldwide [2]. About 85 % of ALS cases are sporadic ALS (SALS) and the remaining 15 % familial (FALS). Mutations in *SOD1* gene are responsible for 20 % of FALS and up to 7 % of SALS cases [3, 4]. Despite a large number of studies, pathogenesis of motor neurons degeneration in ALS is still unclear. Beside the genetic factors, the potential diseases causes include excitotoxicity, deprivation of neurotrophic factors, defects of RNA processing, environmental toxins, oxidative stress and defects in axonal transport along microtubules [5–7]. The latter is crucial for normal function of motor neurons, highly specialized cells with very long axons. The major components of axonal transport are motor proteins, kinesins and dynein/dynactin complexes that move cargo along microtubules [8–10]. Transgenic mice that express human SOD1 gene with G93A mutation develop ALS-like symptoms and are used as a classic model for the disease studies [11–13]. Also the Cra1 mice, with point mutations in the cytoplasmic dynein 1 heavy chain (*Dync1h1*), demonstrate symptoms of motor neuron degeneration and impaired dynein/dynactin mediated retrograde transport [14, 15]. Cytoplasmic dynein is a huge, multi-subunit protein complexes. Its heavy chains show ATPase activity, interact with intermediate and light chains to form the cargo-binding complex and bind the whole complex to microtubules [16, 17]. For its full functional activity dynein requires dynactin, another large, multi-protein complex [18, 19]. The largest dynactin subunit (DCTN1 or p150^Glued^) encoded by *DCTN1* gene binds directly to dynein and microtubules what allows dynein to traverse the microtubule net over long distances. A mutation in the p150 subunit was linked to ALS with vocal cord paresis in humans [20]. The smallest dynactin subunit (DCTN3 or p24) encoded by *DCTN3* gene is probably involved in protein–protein interactions and thus participate in assembly of the dynactin complex [21]. Defects in the function of dynein/dynactin complex are causally linked to multiple neurodegenerative diseases but their molecular mechanism is not clear [22–24]. Previously we indicated affected expression of N-kinesins (involved in the anterograde transport) and C-kinesin (participating in the retrograde transport) in the CNS of sporadically and genetically determined motor neuron degeneration in humans and transgenic mice [25, 26]. In the present work we focused on dynein–dynactin mediated transport studying dynactin heavy (DCTN1) and light (DCTN3) subunits mRNA and protein expression and localization in the motor and sensory brain cortex, hippocampus and spinal cord obtained from autopsy of SALS human subjects, and Dctn1 and Dctn3 mRNA expression in mice with human SOD1G93A (SOD1/+), *Dync1h1* (Cra1/+) and double mutation (Cra1/SOD1) at the presymptomatic and symptomatic stages.

## Experimental Procedure

### Human Tissues

The human material included samples of motor and sensory brain cortex, and cervical spinal cord. The tissues were obtained from autopsy of five SALS and five control subjects with no neurodegenerative disease (Table 1). Autopsies were performed within 72 h from death. The SALS patients were diagnosed at the Department of Neurology, Medical University of Warsaw. They fulfilled the El Escorial criteria for ALS and died in the course of clinically and morphologically definite sporadic ALS. The disease duration ranged from 4 months to 9 years (Table 1).

**Table 1:** Clinical profiles of SALS patients and control subjects

Cases with SALS					Control cases			
No	Sex	Age, years	Disease duration	Type of SALS	No	Sex	Age, years	Cause of death
1	F	76	9 years	Classic	A	F	87	Heart infarction
2	F	76	3 years	Classic	B	F	63	Acute lymphatic leukemia
3	F	58	2 years	Classic	C	M	61	Acute heart failure
4	M	48	4 months	PBP^a^	D	M	49	Acute myeloid leukemia
5	M	63	6 months	Classic	E	F	63	Heart infarction

^a^Progresive Bulbar Palcy

#### RT-qPCR

Total RNA isolated by Chomczynski and Sacchi method [27] was reverse transcribed to a single-stranded cDNA according to the manufacturer’s protocol (Invitrogen, USA). Quantitative gene expression assay was performed using TaqMan probes on the StepOnePlus apparatus (Applied Biosystems), as indicated earlier **(**Kuźma-Kozakiewicz et al., 2012). Expression of *DCTN1* and *DCTN3* was normalized against the housekeeping genes (B2 M and GusB) (Table 2). Measurements of mRNA expression were based on four independent reactions for each cDNA sample. The 120 ng and 480 ng of cDNA were used in duplicates. The results were calculated with normalization of Ct values to the mean Ct value for the reference genes. The relative changes in genes expression were analyzed using the ΔCt method. Statistical analysis was performed using GraphPad Prism (GraphPad Software, San Diego, CA, USA). Results were expressed as mean ± SEM and the data were analyzed by Student’s and Mann–Whitney U tests. Means were considered statistically significant at *p* < 0.05.

**Table 2:** TaqMan probes used in the studies

Gene Symbol	Gene name	Accession no.	Assay ID
B2 M	Beta-2-microglobulin	NM_009735	Hs99999907_m1
GusB	Glucuronidase, beta	NM_010368	Hs99999908_m1
DCTN1	Dynactin 1	NM_001135040	Hs00896387_m1
DCTN3	Dynactin 3	NM_007234	Hs00989657_m1

#### Western Blotting

Samples of freshly frozen human tissues were homogenized in isotonic PBS, centrifuged (20 min, 12,000×g, 4 °C), and the obtained supernatant was used for further studies. Western blotting was performed after electrophoresis in polyacrylamide gel according to Laemmli [28], with rabbit polyclonal DCTN1 (H-300) antibodies (Santa Cruz Biotech; 135–150 kDa) and goat polyclonal DCTN3 antibodies (Abcam; 20–21 kDa). Mouse monoclonal anti-beta-actin antibody (Sigma) was used as control. Blots were visualized using Pierce^®^ ECL Western Blotting Substrate (Thermo Sci.). System UVI-KS4000 (Syngen Biotech.) was used for densitometric analysis of the results.

#### Immunohistochemical Study

Formalin-fixed and paraffin-embedded tissue samples from the primary motor cortex (precentral gyrus), primary sensory cortex (postcental gyrus) and spinal cord of both SALS and control cases were used. The immunohistochemical reactions with primary antibodies against DCTN1 (1:100; Santa Cruz Biotech.) and DCTN3 (1:500; Atlas) were performed according to streptavidin–biotin peroxidase method. A routine microwave pretreatment of antigen retrieval (boiling in 10 mM citrate buffer, pH 6.0) was performed before incubation (1 h, room temperature) with the primary antibodies against DCTN3. Afterwards the sections were incubated with the secondary antibodies (Rabbit ABC Staining System, Santa Cruz Biotech.) followed by 3,3′-diaminobenzidine. The examined material was evaluated at light microscopy (Nikon) and the numerical aperture of the lens was 40.

### Mouse Tissues

The studies were conducted on mouse strain C57BL/6GJ-C3H/HeJ (B6-C3H) hybrids with human SOD1G93A mutation (SOD1/+), with dynein heavy chain 1 mutation (*Dync1h1*; so-called Cra1/+), with double *Dync1h/*SOD1G93A mutation (Cra1/SOD1), and genetic background- and age-matched wild-type controls (+/+). The hSOD1G93A males (B6 background; Jackson Laboratories, Barr Harbor, ME, USA) were crossed with Cra1/+ heterozygote females (C3He background; Ingenium Pharmaceuticals AG, Martinsried, Germany) [29]. There were 6 males in each animal group. Mice were at the presymptomatic stage without symptoms of neurodegeneration and at the symptomatic end stage. The presymptomatic SOD1/+ and Cra1/SOD1mice were 70 days old, while the Cra1/+ mice—70 and 140 days old. The symptomatic SOD1/+ and Cra1/SOD1mice were 140 days old and Cra1/+ mice—365 days old.

#### RT-PCR

The expression of dynactin heavy (Dctn1) and light subunits (Dctn3)-mRNA was studied in mouse brain frontal cortex, cervical spinal cord and hippocampus included as a control structure. Total RNA was isolated with the use of NucleoSpin^®^ RNAII kit (Macherey–Nagel) according to manufacturer’s protocol followed by reverse transcription, polymerase chain reaction and DNA electrophoresis in agarose gel. Specific oligonucleotide primers for mouse dynactin Dctn1 were: 5′-TGCTGCTCCAGGAGAGGTGA and 5′-CCTAAGAAGGCACCGACAGC, and for dynactin light chain (Dctn3) were as follows: 5′-GGCCTCGAAGTTGCAGTTCA and 5′-CTCTGCTGGCTTCACTTGCT. The primers for ribosomal S12 protein RNA band (Rps12, housekeeping gene, internal control) were: 5′-TCGCATCCAACTGTGATGAG and 5′-TCTTTGCCATAGTCCTTAACCACTACG. RNA level was measured in semi-quantitative way and expressed as the ratio of the optical density band of studied dynactin to the optical density of ribosomal S12 protein RNA. Each assay was performed in duplicates and repeated two times. System UVI-KS4000 (Syngen Biotechnology) was used for densitometric analysis. Results were expressed as mean ± SD and the data were analyzed by the two-way analysis of variance (ANOVA). Quantitative comparison between studied groups was performed by Student’s *t* test using Statistica 9.0 (StatSoft Inc, USA). Means were considered statistically significant at *p* < 0.05.

#### Ethics Statement

The studies on human tissues were approved by the Bioethic Committee at the Medical University of Warsaw, Poland (KB/253/2003) in accordance with Declaration of Helsinki. Non of the patients (while still alive) or their relatives (after the patient’s death) disagreed on the autopsy and tissue studies. The studies on mice tissues were approved by the Ethic Committee for Experiments on Animals at the Medical University of Warsaw, Poland (no AO-KEZ/622/3).

## Results

### Dynactin in Human CNS

**Fig. 1:**
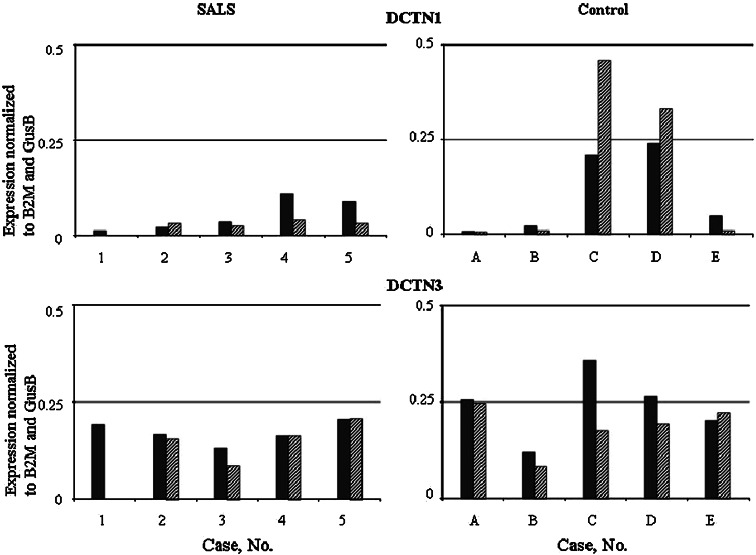
Expression of dynactin DCTN1 and DCTN3 mRNA in the CNS of SALS and control cases. The expression was studied by real-time qPCR, as described in the Material and method section. The results were quantified as the ratio of studied dynactin (DCTN1 or DCTN3) expression to the expression of housekeeping genes (B2M and GusB; ΔCt method). No. 1–5, cases with SALS; *a*–*e*, control cases. *Black bars* motor cortex; *striped bars* sensory cortex

Expression of DCTN1-mRNA in the motor cortex of individual SALS brains was higher than in the corresponding SALS sensory cortex (Fig. 1). The mean DCTN1-mRNA expression was slightly higher in SALS motor than SALS sensory cortex (Table 3). However, in SALS brains expression of DCTN1 in both types of cortex and also in the spinal cord, was lower than in control tissues (Table 3).

**Table 3:** Mean dynactin subunits expression in various parts of human CNS

Part of CNS	Cases	DCTN1	DCTN3
Motor cortex	Control	0.104 ± 0.049	0.240 ± 0.038
	SALS	0.052 ± 0.019	0.171 ± 0.013
Sensory cortex	Control	0.162 ± 0.096	0.184 ± 0.028
	SALS	0.031 ± 0.003	0.153 ± 0.025
Spinal cord	Control	0.093 ± 0.049	0.115 ± 0.028
	SALS	0.045 ± 0.006	0.056 ± 0.005

The DCTN3-mRNA expression in the motor and sensory cortex of individual SALS cases was at the same level (Fig. 1). The mean expression of DCTN3-mRNA was similar in SALS motor and SALS sensory cortex but slightly lower than in control cortexes. It was also lower in SALS spinal cord compared to the control tissue (Table 3). All changes were statistically not significant.

**Fig. 2:**
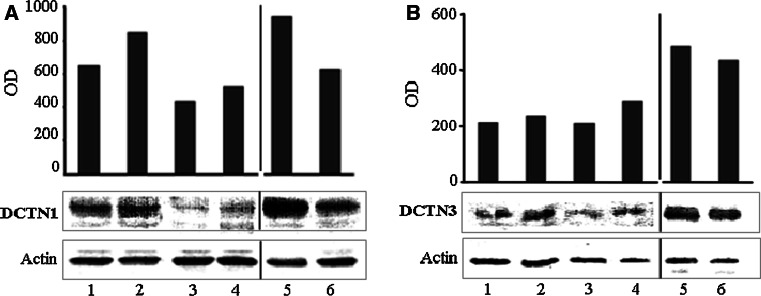
Expression of dynactin DCTN1 and DCTN3 protein in the CNS of SALS and control cases. The expression was studied by Western blotting, as indicated in the Material and method section. Comparable amounts of protein (20 μg for DCTN1 and 40 μg for DCTN3) from representative control (no D) and SALS (no 4) brains were run in each lane. A; DCTN1: *line 1* control motor cortex (optical density: OD 651), *line 2* control sensory cortex (OD 847), *line 3* SALS motor cortex (OD 439), *line 4* SALS sensory cortex (OD 519), *line 5* control spinal cord (OD 940), *line 6* SALS spinal cord (OD 627). **b** DCTN3: *line 1* control motor cortex (OD 211), *line 2* control sensory cortex (OD 238), *line 3* SALS motor cortex (OD 210), *line 4* SALS sensory cortex (OD 290), *line 5* control spinal cord (OD 180), *line 6* SALS spinal cord (OD 92)

In SALS motor cortex the level of DCTN1 protein was lower than in SALS sensory cortex, and lower than in corresponding control cortexes (Fig. 2). It was also lower in SALS spinal cord than in the control tissue (Fig. 2). The level of DCTN3 protein was similar in both parts of brain cortex, and there were no differences between SALS and control cases (Fig. 2). In the spinal cords, the level of DCTN3 protein was much lower in SALS than in the control tissues (Fig. 2).

**Fig. 3:**
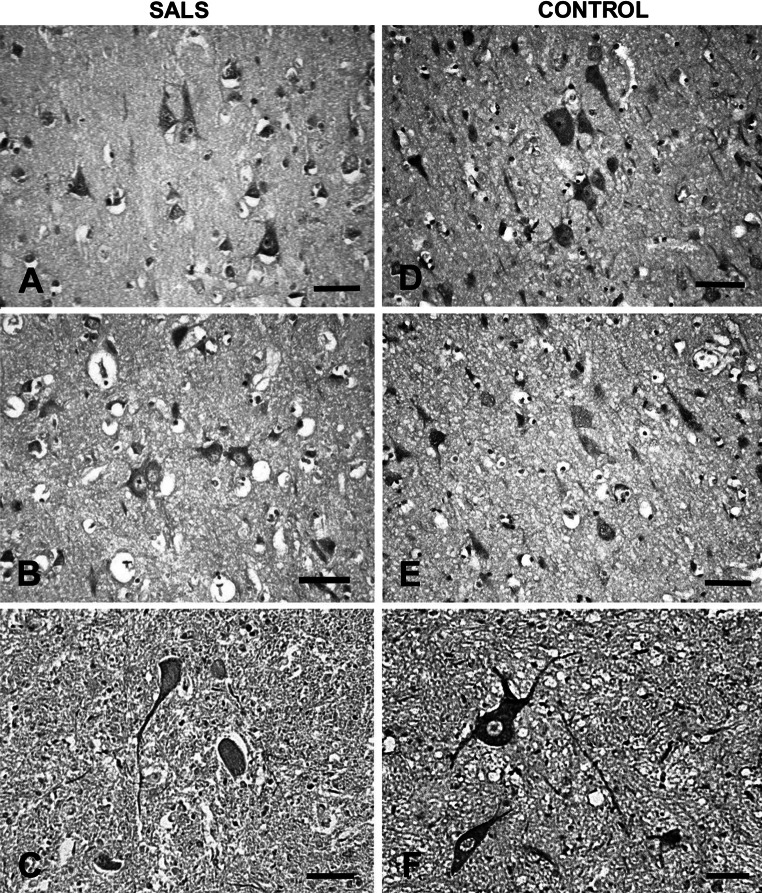
Representative immunohistochemistry for dynactin DCTN1 on sections from SALS and control human cases. **a** Immunopossitive neurons and their processes in SALS motor cortex and visible moderate loss of neuronal cells; **b** positive immunoreactivity in SALS neurons and axons in the sensory cortex; **c** moderate immunoexpression in pericaria and axons of the preserved SALS anterior horn motoneurons with features of degeneration; **d** pronounced immunoreactivity of control neuronal processes and pericaria in the motor cortex; **e** positive immune reaction of different intensity in control axons and neuronal pericaria in the sensory cortex; **f** very strong immunoreactivity of processes and motoneuron pericaria in control spinal cord anterior horn. *Bars* 100 μm each

**Fig. 4:**
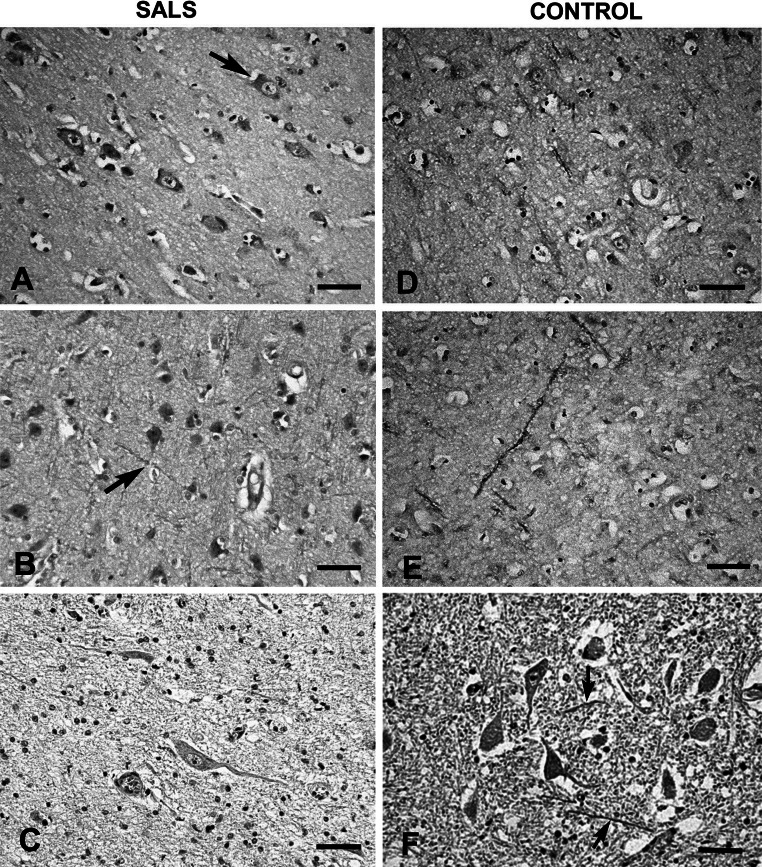
Representative immunohistochemistry for dynactin DCTN3 on sections from SALS and control human cases. **a** Weakly immunopositive single neuron in SALS motor cortex (*arrow*) and visible loss of neurons; **b** mostly negative immune reaction in SALS neurons and axons (*arrow*) in the sensory cortex; **c** very poor or absent immunoexpression in the preserved motoneurons with features of degeneration in the anterior horn of SALS spinal cord; **d** weak immunolabel in pericaria of neurons and pronounced in their processes in the control motor cortex; **e** strong immunoreactivity of axons and poor of neuronal pericaria in the control sensory cortex; **f** weak immunoexpression in control pericaria of anterior horn motoneurons and mild in axons (*arrows*). *Bars* 100 μm

Morphological examination revealed degeneration and loss of neurons in SALS—severe in the anterior horns of the spinal cord and mild in the motor cortex while the sensory cortex was relatively intact. In immunohistochemistry DNCT1 protein was seen in pericaria, axons, and dendrites of neurons, and was less intense in SALS than in the control material (Fig. 3a–f). The difference in DNCT1 expression was especially evident in the anterior horns of individuals with SALS (Fig. 3c, f) where the majority of preserved motoneurons revealed mild immunoreactivity in cell bodies and weak or sometimes even absent immunoreactivity in neuronal processes. Immunoexpression of DNCT3 in SALS was poor in all examined cases and in all CNS structures. In the motor and sensory cortex only single neurons demonstrated weak immunolabel of cell pericarion while in the control material the immunoreactivity was much stronger and visible mainly in neuronal processes (Fig. 4a–e). The same phenomenon was observed in the anterior horn motoneurons (Fig. 4c, f).

### Dynactin in Transgenic Mice CNS

**Fig. 5:**
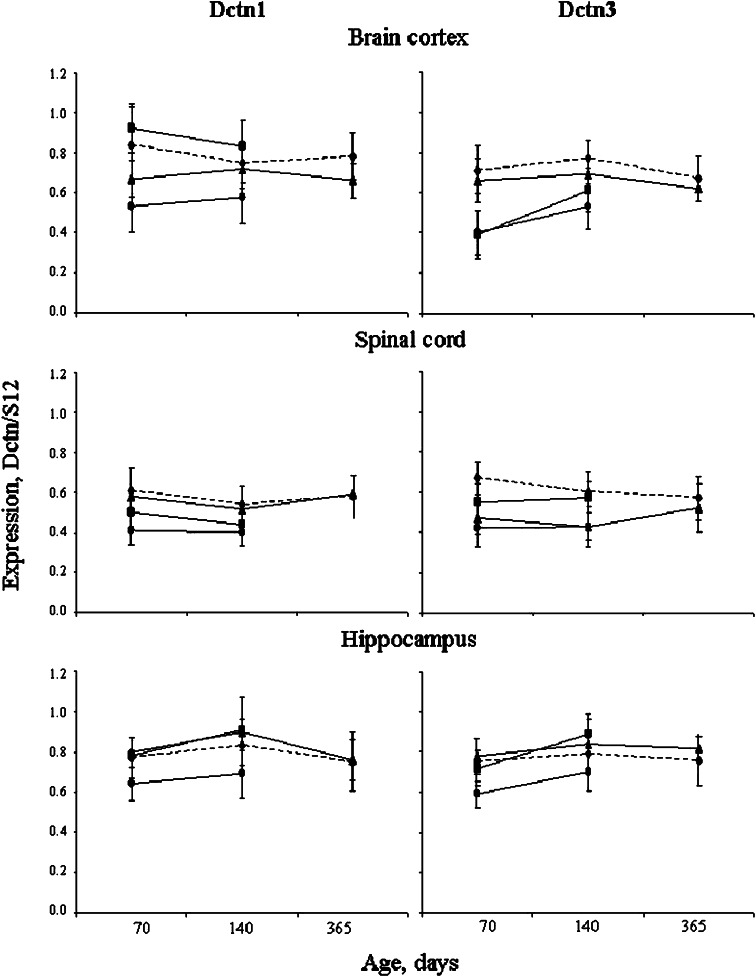
Expression of dynactin Dctn1 or Dctn3 mRNA in the CNS of transgenic mice. The expression was determined by RT-PCR and expressed as the ratio of the optical density (OD) value of Dctn1 or Dctn3 to the optical density of S12 protein RNA, as indicated in the Material and Method section. *Filled diamond dashed lines* wild-type controls (+/+); *filled triangles* Cra1/+ mice; *filled circles* SOD1/+ mice; *filled squares* Cra1/SOD1 hybrides

In SOD1/+, but neither in Cra1/SOD1 nor Cra1/+ mice, the expression of dynactin subunits Dctn1- and Dctn3-mRNA was significantly decreased in all studied parts of the CNS compared to the age-matched wild type controls (Fig. 5; Table 4). In the frontal brain cortex of SOD1/+ mice the expression of each subunit was lower at the presymptomatic (age 70 days) than at the symptomatic stage (age 140 days). When compared to the control tissues, Dctn1 expression was decreased to about 65 % (*p* < 0.0001) and 80 % (*p* < 0.005) in presymptomatic and symptomatic mice, respectively, and that of Dctn3 to 55 % (*p* < 0.0001) and 70 % (*p* < 0.001), respectively (Table 4). In Cra1/SOD1 hybrids the expression of Dctn1-mRNA was slightly but not significantly increased compared to the control groups, whereas that of Dcnt3 was decreased to 55 % (*p* < 0.0001) and 80 % (*p* < 0.005) at the presymptomatic and the symptomatic stage, respectively (Table 4; Fig. 5). In Cra1/+ mice the expression of Dctn1-mRNA was slightly but significantly lower at both stages (age 70 days, *p* < 0.01, age 356 days, *p* < 0.0.01), whereas that of Dctn3 was practically unchanged (Fig. 5).

**Table 4:** Percentage of dynactin subunits expression in the CNS of transgenic mice

		Dctn1			Dctn3		
Part of CNS	Clin. stage	SOD1/+	Cra1/SOD1	Cra1/+	SOD1/+	Cra1/SOD1	Cra1/+
Remaining expression, %							
Frontal cortex	Presympt.	65*	110	80*	55*	55*	90
	Sympt.	80*	110	85*	70*	80*	90
Spinal cord	Presympt.	65*	80	95	60*	80	70*
	Sympt.	75*	80	100	70*	95	90
Hippoc.	Presympt.	85*	100	105	80*	95	105
	Sympt.	85*	110	100	90*	110	110

All presymptomatic mice were at the age of 70 days, symptomatic SOD1/+ and Cra1/SOD1 at the age of 140 days, and symptomatic Cra1/+ at the age of 365 days. The values were calculated in comparison with the age-matched wild-type control mice, taken as 100 %

* Data statistically significant when calculated for the expression level

In the spinal cord of SOD1/+ mice the expression of both dynactin subunits was lower at the presymptomatic stage (*p* < 0.001 for each subunit) than at the symptomatic stage (*p* < 0.0001 for Dctn1 and *p* < 0.001 for Dctn3) (Table 4). In Cra1/SOD1 mice the dynactins expression was slightly decreased when compared to the control animals but the changes were not significant (Fig. 5). However, when compared to SOD1/+ mice, the expression of Dctn1 was significantly higher at the presymptomatic stage (*p* < 0.005), and that of Dctn3 was higher in both stages (*p* < 0.0005 and *p* < 0.0001, respectively). In all Cra1/+ mice Dctn1-mRNA was unchanged and Dctn3 significantly lower than in control group only at the presymptomatic stages (age 70 and 140, *p* < 0.001) (Table 4, Fig. 5).

In the hippocampus of SOD1/+ mice expression of each subunit was slightly but significantly lowered at both stages (*p* < 0.0005 and *p* < 0.01 for Dctn1, *p* < 0.0001 and 0.001 for Dctn3, at the presymptomatic and symptomatic stage, respectively). In Cra1/SOD1 and Cra1/+ mice expression of both dynactin subunits was unchanged (Fig. 5).

Analysis of variance showed that Dctn1-mRNA and Dctn3-mRNA expression was dependent on both the mutation and the clinical stage only in the frontal cortex [F(3.86) = 2.98, *p* = 0.036; F(3.87) = 6.91, *p* = 0.0003, respectively].

## Discussion

Dynein/dynactin complex is involved in axon maintenance, removal of damaged organelles, vesicles, misfolded and aggregated proteins from axons to the cell body [30]. Many point mutations in the gene encoding for dynactin were found in sporadic and familial cases of ALS [31, 32]. The largest dynactin subunit (DCTN1, called also p150^Glued^) which mediates dynein–dynactin interaction is a critical component of the whole dynein/dynactin complex. It was found that a single base-pair change leading to a glycine-59-serine (p.G59S) substitution in glycine-rich domain of this subunit accelerated motor neuron degeneration in humans and mice which resembled ALS [33, 34]. Vilarino-Guell et al. [35] after sequencing all *DCTN1* exons and exon–intron boundaries in 286 samples diagnosed with neurodegenerative diseases (PD, FTLD, ALS) concluded that pathogenic mutations in DCTN1 are rare. Lately Fujiwara and Morimoto [7] discovered that overexpression of p150^Glued^ and microtubule stabilization cooperatively suppress axon degeneration.

In the present study we found that in individual SALS subjects, DCTN1-mRNA expression was higher in the motor than in the sensory brain cortex. In our earlier studies conducted on the same SALS subjects, we found that kinesins-mRNA expression was also higher in the motor cortex [25]. In the control brain cortexes (without neurodegenerative disorders), the situation was opposite—DCTN1 and kinesins-mRNA expression was lower in the motor compared to the sensory cortex. However, the increase of DCTN1-mRNA in SALS was not followed by the increase of protein, the level of which in the motor cortex was even lower than in the sensory cortex. This may indicate that in the motor cortex, the structure usually affected by the process of degeneration, the effort is made to restore bidirectional axonal transport but it is not sufficient since mRNA is not effectively translated to protein. The highest increase of DCTN1-mRNA and also kinesins-mRNA expression was observed in the subjects with short disease duration (4 months, 2 years). However, the low number of studied brains, due to difficulties obtaining human tissues, makes it impossible to find the correlation between the disease duration and motor proteins expression.

The comparison of SALS and control cases indicated lower values of DCTN1 expression (on the mRNA and protein level) in both SALS cortexes and also in the spinal cord. Those results were supported by immunoexpression, which indicated that DCTN1 protein in the preserved SALS motoneurons revealed mild immunoreactivity and was less intense than in control material—the difference was especially evident in the spinal cord anterior horns. Our results are with agreement with the studies of Ikenaka et al. [36], who observed the reduction of dynactin 1 expression in spinal motor neurons of ALS patients and indicated its connection with the impairment of autophagosomes, a cargo of dynien/dynactin complexes.

Differently to DCTN1, in the brain cortex of SALS cases the expression of DCTN3 was similar in both SALS cortexes and it didn’t differ from the expression in control cortexes (on both mRNA and protein levels). In the spinal cord the expression of DCTN3 was much lower in SALS than in control tissues. In immunohistochemistry DCTN3 was hardly seen in all SALS tissues but was much better visible in controls.

Mutations in the gene encoding SOD1 cause 2–3 % of all ALS cases and induce motor neuron degeneration in transgenic mice [3, 23]. Mutant SOD1 affects bidirectional axonal transport of specific cargoes but the precise mechanism is not fully understood [23]. According to Ligon et al. [37] mutant SOD1 associates with dynein, alteres its cellular localization and consequently the function of dynein/dynactin complex. Zhang et al. [38], indicated that mutant but not normal SOD1 directly interacts with dynein heavy chain forming aggregates. We observed a significant decrease of dynactin heavy and light subunits (Dctn1, Dctn3) expression in mice with human SOD1G93A mutation. Differently than in human SALS cases, where only DCTN1 expression was decreased, in mice the expression of both dynactin subunits was decreased in the frontal brain cortex comparing to wild-type controls. Similar to humans, the expression of studied dynactins was decreased also in the spinal cord. As we showed earlier the expression of kinesins KIF5A and KIF5C involved in the anterograde transport but also the expression of KIFC3 participating in the retrograde transport, was significantly increased in the frontal cortex and the spinal cord of SOD1/+ mice comparing to the corresponding control tissues [25]. Interestingly the changes in dynactin subunits preceded those in kinesins, since the decrease of dynactin subunits expression was observed already at the presymptomatic stage and was more severe than at the symptomatic stage, whereas that of kinesins (including KIFC3) appeared later, at the symptomatic stage. Such differences indicate the existence of a compensatory mechanism aiming to restore affected bidirectional axonal transport. All the more, that dynactin has been implicated not only in the retrograde but also in the anterograde transport, and its large subunit have been shown to interact with particular kinesins [10, 39, 40].

All motor proteins transport their cargoes on tracts formed by microtubules. Microtubules assembly, stabilization and organization into bundles depend on tau protein [41]. Tau also enhances the attachment of dynein/dynactin complex to microtubules by binding the N-terminal domain to the C-terminus of dynactin p150 (Dctn1) subunit [42]. As we have previously shown, tau expression is lower in mice with SOD1 mediated ALS than in wild-type animals due to defective N-terminal alternative splicing [43, 44]. It indicates that dynein/dynactin-dependent transport may be impeded due to tau dysfunction but also, as we have just shown, by significant decrease of dynactin Dctn1 and Dctn3 subunits expression.

Cra1 mice with dynein heavy chain (*Dync1h1)* mutations present phenotype similar but milder than SOD1G93A mice but in contrast to SOD1 mice their life span remains almost unchanged [14, 15, 45]. However, the involvement of the *Dync1h1* mutations in pathogenesis of motor neuron degeneration is controversial [46, 47]. According to Dupuis et al. [48], Cra1 heterozygotes do not display typical feature of motor neuron disease, even in aged animals, but instead they develop an early-onset sensory neuropathy. We found much fewer changes in the expression of motor proteins in the CNS of Cra1/+ than SOD1/+ mice. In Cra1/+ mice we observed a slight decrease of Dctn1-mRNA only in the frontal cortex, and decrease of Dctn3-mRNA only in the spinal cord. Kinesin KIFC3-mRNA expression (involved in retrograde transport) was unchanged in Cra1/+ mice, but that of N-kinesins (anterograde transport) was raised, especially at the symptomatic stage [26]. There were also clear differences between tau expression and its alternative splicing in mice with *Dync1h1* and SOD1G93A mutation [44]. All those results indicate that the axonal transport may be affected in Cra1/+ mice due to dysfunction of the axonal motors and the structure of cytoskeleton but not as severely as in SOD1/+ mice.

In mice with double SOD1G93A and *Dync1h1* mutations defective retrograde transport is partially corrected and the life span of SOD1 mice extended [45]. According to Fergani et al. [49] increased survival of double heterozygotes may be mediated by a reversal in energy deficit and increased IGF-1 availability. The *Dync1h1* mutation also attenuates motor neuron degeneration in SOD1G93A mice [29]. Zhang et al. [38] suggested that mutant SOD1 shows very low affinity for mutant dynein (in Cra1 and Loa mice) and thus the protein aggregation and SOD1 toxicity in double transgenic animals may be reduced. Although the underlying mechanisms of its protection are not clear, we observed fewer changes in the expression of both dynactin subunits, and also kinesins in the CNS of Cra1/SOD1 mice comparing to SOD1/+ , and more changes comparing to Cra1/+ transgenics [26]. In the spinal cord of Cra1/SOD1 presymptomatic mice both dynactins expression was significantly higher than in SOD1/+ mice and that of Dctn3 was higher also at the symptomatic stage. In concordance with other authors [29, 45, 49] our results confirm that the double Cra1/SOD1 mutations protects axonal transport which in the spinal cord seems to be relatively normal. It may be worth noticing that in the hippocampus, the structure included into our studies as a control one, a slight decrease of both dynectin subunits expression was observed in SOD1/+ mice, whereas there were no changes either in Cra1/+ or in Cra1/SOD1 transgenics.

It can be concluded that in SALS and SOD1-related ALS the impairment of dynein-mediated retrograde axonal transport and degeneration of motor neurons may be related to dynactin subunits deficiency and subsequent disruption of the whole dynein/dynactin complex structure and function. The *Dync1h1* mutation itself has slight negative effect on dynactin subunits expression but positively affects the changes caused by SOD1G93A mutation.

## Abbreviations

ALS: Amyotrophic lateral sclerosis
SALS: Sporadic amyotrophic lateral sclerosis
AD: Alzheimer’s disease
FTD: Frontotemporal dementia
FTLD: Frontotemporal lobar degeneration
PD: Parkinson’s disease
MND: Motor neuron degeneration
CNS: Central nervous system
DCTN1: Dynactin heavy chain
DCTN3: Dynactin light chain
hSOD1: Human copper–zinc superoxide dismutase 1
Dync1h1: Cytoplasmic dynein 1 heavy chains
Cra1: Cramping 1
KIF: Kinesin
PBP: Progressive bulbar palsy
